# Retinal Ganglion Cells are Resistant to Photoreceptor Loss in Retinal Degeneration

**DOI:** 10.1371/journal.pone.0068084

**Published:** 2013-06-28

**Authors:** Bin Lin, Edward Bo Peng

**Affiliations:** 1 Department of Anatomy, The University of Hong Kong, Li Ka Shing Faculty of Medicine, Pokfulam, Hong Kong; 2 Department of Ophthalmology, The University of Hong Kong, Li Ka Shing Faculty of Medicine, Pokfulam, Hong Kong; 3 State Key Laboratory of Brain and Cognitive Sciences, The University of Hong Kong, Li Ka Shing Faculty of Medicine, Pokfulam, Hong Kong; University of Florida, United States of America

## Abstract

The rapid and massive degeneration of photoreceptors in retinal degeneration might have a dramatic negative effect on retinal circuits downstream of photoreceptors. However, the impact of photoreceptor loss on the morphology and function of retinal ganglion cells (RGCs) is not fully understood, precluding the rational design of therapeutic interventions that can reverse the progressive loss of retinal function. The present study investigated the morphological changes in several identified RGCs in the retinal degeneration *rd1* mouse model of retinitis pigmentosa (RP), using a combination of viral transfection, microinjection of neurobiotin and confocal microscopy. Individual RGCs were visualized with a high degree of detail using an adeno-associated virus (AAV) vector carrying the gene for enhanced green fluorescent protein (EGFP), allowed for large-scale surveys of the morphology of RGCs over a wide age range. Interestingly, we found that the RGCs of nine different types we encountered were especially resistant to photoreceptor degeneration, and retained their fine dendritic geometry well beyond the complete death of photoreceptors. In addition, the RGC-specific markers revealed a remarkable degree of stability in both morphology and numbers of two identified types of RGCs for up to 18 months of age. Collectively, our data suggest that ganglion cells, the only output cells of the retina, are well preserved morphologically, indicating the ganglion cell population might be an attractive target for treating vision loss.

## Introduction

The eye provides vision that relies on the integrity of cellular structures and functions of the retina. Like many other portions of the central nervous system (CNS), the retina is subject to a variety of inherited and acquired degenerative conditions [1], [2]. The primary pathological event in many forms of retinal degeneration is the degeneration of photoreceptor cells, which serve to initiate the process of vision by converting light into neural signals. One of the most common retinal degenerations is retinitis pigmentosa (RP). RP is a group of inherited human diseases characterized by progressive degeneration of photoreceptor cells and loss of photoreceptor function, eventually leading to functional blindness [3]–[7]. To date, patients with retinal degeneration, such as RP and age-related macular degeneration (AMD), have few possibilities for therapy. Understanding the pathophysiology of retinal degeneration and the accompanying changes in the cellular architecture of the retina is thus crucial for designing rational therapeutic interventions to rescue vision.

The integrity of second- and third-order retinal neurons and their ability to reliably process and transmit visual signals to the brain is essential for many vision rescue strategies under development, such as photoreceptor replacements by injecting either immature post-mitotic rods or engineered stem cells [8], [9]. Ultimately, the success of any of these approaches depends on the functional integrity of retinal ganglion cells (RGCs), the only projection neuron of the retina, whose axons carry visual information to visual processing centers in the brain. It has become increasingly clear that a negative impact of photoreceptor loss on the remaining retina should be expected. Indeed, a growing body of evidence over last decades suggests that the secondary remodeling in the remaining retinal neurons, such as bipolar cells and horizontal cells, occurs during retinal degeneration in *rd1* mouse retinas of RP models [10]–[13]. Moreover, retinal neurons in *rd1* mice are reported to undergo neurochemical plasticity as well, which occur even prior to anatomical remodeling [14]. However, the impact of photoreceptor loss on RGC morphology is not completely understood in *rd1* mouse retinas. Postmortem studies in aged human patients with RP shows a mild to severe RGC loss in retinas [15], [16]. However, estimates of RGC survival in animal models of RP have led to somewhat conflicting results. RGC loss has been reported for rd1 mice and P23H and RCS rats [17]–[20], while high preservation of RGCs in rd1 and rd10 mice is observed in other study [21]–[23]. Furthermore, functional studies have shown that RGCs appear to preserve the intrinsic membrane and firing properties in the rd1 mutant mouse [24].

In the present study, we investigated the morphological properties of RGCs in the retinal degeneration *rd1* mouse model of RP, using a combination of viral transfection, microinjection of neurobiotin and confocal fluorescence microscopy. The *rd1* mouse carries a non-sense mutation in exon 7 of the beta subunit of rod photoreceptor phosphodiesterase gene with an early onset and rapidly progressing degeneration of photoreceptors [4], [5], [7], as occurs in a small portion of human RP patients [25]. Here, we explored the feasibility of analyzing individual RGCs by using an adeno-associated virus (AAV) vector carrying the gene for enhanced green fluorescent protein (EGFP). This approach visualized morphologies of individual RGCs with a high degree of detail, which allowed for large-scale surveys of neuronal morphology of RGCs in *rd1* mice over a wide age range. In addition, photoreceptor degeneration shows a central-to-peripheral temporal progression pattern across the retinal surface of *rd1* mice [26]–[29], so do rod bipolar and cone bipolar cells [13], [30]. Degeneration of bipolar cells will have direct effect on RGCs, since RGCs receive synaptic inputs directly from cone bipolar cells. To investigate whether RGC followed the same temporal progression landmark in *rd1* mice, we applied RGC-specific markers to study the number and morphology of two identified RGCs across the retinal surface.

## Materials and Methods

### Animals

Experiments were carried out on wild type C57BL/6J mice and C3H/HeJ mutant mice, homozygous for *rd1* mutation (*rd/rd*) (The Jackson Laboratory, USA) at the ages of post-natal day 30 (P30) to 18 months. Animals were housed on a 12-hour light-dark cycle and maintained at the Laboratory Animal Unit, The University of Hong Kong. All experimental procedures were approved by the Committee on the Use of Live Animals in Teaching and Research at The University of Hong Kong and conducted in accordance with the ARVO statement for the use of animals. The Laboratory Animal Unit of the University of Hong Kong is fully accredited by the Association for Assessment and Accreditation of Laboratory Animal Care International (AAALAC international).

### Construction of Adeno-associated Viral (AAV) Vectors

For making AAVs, the cDNA of GFP was inserted downstream of the hybrid cytomegalovirus (CMV) immediate early enhancer/chicken β-actin (CBA) enhancer in the plasmid pAAV-MCS, which contained the AAV2 inverted terminal repeats and a human growth hormone polyA signal. The helper plasmid (Stratagene) that encoded E2A, E4 and VA and pAAV-RC (Stratagene) that encoded the AAV2 genes (rep and cap) were used for co-transfection in 293T cells to generate recombinant AAV. The vectors were then packaged, purified and concentrated in PBS at titer in the range of 2.3–7.5×10^12^ genome copies/ml.

### Intravitreal Injections of Viral Vectors

Animals were anesthetized with a mixture of ketamine hydrochloride (30–40 mg/kg) and xylazine (3–6 mg/kg). With the aid of an operating microscope, a small incision was made in the sclera, and 0.5–1 µl of vector suspension was slowly injected through the incision into the ganglion cell side of the retina to transfect RGCs, using a 5 µl syringe (Hamilton) equipped with a 33-gauge blunt needle. Following intravitreal delivery, antibiotics (bacitracin/neomycin/polymyxin) were applied to the eyes. The animals were observed daily for evidence of inflammation. None was observed.

### Intracellular Injections of Neurobiotin

Intracellular injections were performed following the methods previously described [31], [32]. Briefly, retinas were isolated from posterior eyecups in carboxygenated Ames medium (Sigma, St. Louis, MO). Using a 40X water immersion objective (Achroplan, NA 0.75, Zeiss), RGCs prelabeled with the fluorescent dye Acridine Orange (Sigma, St. Louis, MO) were penetrated with sharp pipettes, whose resistance was more than 150 MΩ. The electrode tips were filled with a solution of 0.5% Lucifer Yellow (Sigma) and 4% Neurobiotin (Vector Laboratory, Burlingame, CA) in 0.1 M Tris/HCl buffer, pH 7.6, and back-filled with 3 M lithium chloride. Neurobiotin was iontophoresed with a positive current of 0.5–0.8 nA for 5 to 10 minutes. After the last injection, retinas were fixed in 4% formaldehyde in phosphate-buffered saline (PBS; pH 7.4) for 30 minutes, washed in PBS containing 0.05% Triton X-100, 0.1% sodium azide. Then, retinas reacted 1 day at room temperature with Alexa 594-streptavidin conjugate (1∶100; Invitrogen-Molecular Probes) in PBS. Following several washes in PBS, retinas were incubated in diamidinophenylindole (DAPI, Sigma, St. Louis, MO) for 1 hour. All retinas were then washed 3×10 minutes in PBS and mounted in Vectashield (Vector Laboratories, Burlingame, CA) and observed immediately.

### Immunocytochemistry and Confocal Imaging

The animals were deeply anesthetized with the mixture of ketamine hydrochloride and xylazin at different time points following the viral injections. Eyes were quickly enucleated after a reference point was taken to label the superior pole and the retinas were dissected free of the vitreous and sclera in carboxygenated Ames’ Medium (Sigma, St. Louis, MO), and then fixed in 4% paraformaldehyde (PFA) for 0.5–1 hour. Some of retinas were sectioned serially at a thickness of 10–12 µm on a cryostat. Both whole-mounted retinas and cross sections were blocked in a solution containing 3% normal goat serum (NGS), 1% bovine serum albumin (BSA), and 0.3% Triton X-100 in PBS for 1 hour. All primary antibodies used for this study are listed in Table 1. The primary antibodies were diluted with a blocking solution (1% NGS, 1% BSA, 0.1% Triton X-100 in PBS) and applied from overnight to 5 days at 4°C. After blocking and rinsing, a secondary antibody conjugated to either Alexa 488 (1∶500; Molecular Probes, Eugene, OR) or Alexa 594 (1∶500; Molecular Probes, Eugene, OR) was applied for 2 hours at room temperature. In double-labeling experiments using primary antibodies from different hosts, primary antibodies were applied simultaneously and then visualized by application of appropriate secondary antibodies. Sections and whole-mounted retinas were rinsed, cover slipped in Vectashield mounting medium (Vector Laboratories, Burlingame, CA). To reveal detailed morphology of both SMI32 and melanopsin positive cells, retinas were reacted with biotinylated goat-anti-rabbit secondary antibody after application of a primary antibody, SMI32 or melanopsin. After several washes in PBS, retinas were then reacted with ABC reagent (Vector labs, Burlingame, CA) for 4 hours at room temperature and developed using Diaminobenzidine (DAB) reagent (Vector Labs).

**Table 1:** Primary Antibody Information.

Antigen	Immunogen	Manufacturer, type of antibody	Dilution
Green fluorescen protein (GFP)	GFP isolated from the jellyfish Aequorea victoria	Invitrogen, USA; Rabbit polyclonal	1∶500
Neurofilaments	Purified neurofilaments	Covance, USA; Mouse monoclonal #SMI-32	1∶200
Melanopsin	Synthetic peptide corresponds to amino acid residues 1–19 from rat melanopsin protein	Fisher Scientific, rabbit polyclonal	1∶500
CD90.2	Mouse thymus or spleen	BD Bioscience, monoclonal	1∶100

Confocal micrographs of fluorescent specimens from retinal flat-mounted preparations and vertical sections were captured using a Zeiss LSM 700 Meta Axioplan 2 laser scanning confocal microscope (Carl Zeiss) equipped with argon and helium-neon lasers. Plan-Apochromat 63x/1.4 or 40x/1.4 oil immersion objective lenses were used. Images scale was calibrated, and if necessary, brightness and contrast were adjusted using Photoshop CS3 software (Adobe Systems, San Jose, CA, USA).

### Antibody Characterization

Refer to Table 1 for a list of all primary antibodies used. The Green Fluorescent Protein (GFP) antibody is IgG fraction from rabbit serum raised against GFP. The PA1–780 melanopsin antibody detects ∼53 and ∼85 kDa proteins, representing over-expressed unglycosylated and glycosylated melanopsin from HEK 293 cells, and has been successfully used in Western blot, immunofluorescence, and immunocytochemistry procedures. The specificity of the antibodies was confirmed in immunoblot experiments with melanopsin expressed in HEK 293 cells. PA1-780 labels retinal ganglion cells in mouse and rat retinas [33]. SMI32 reacts with a non-phosphorylated epitope in neurofilament H of most mammalian species. Immunocytochemically, SMI32 visualizes neuronal cell bodies, dendrites and some thick axons in the central and peripheral nervous systems. SMI32 is relatively selective for α ganglion cells in the mouse retina [32], [34]. CD90.2 is a 25–35 kD immunoglobulin superfamily member also known as Thy-1.2, a glycosylphosphatidylinositol (GPI)-anchored membrane glycoprotein. CD90.2 has been shown to be a marker for differentiated retinal ganglion cells in the mouse [32], [35].

### Data Analysis

Quantification of RGCs was conducted in retinal whole-mounts, using a 40× objective lens (numerical aperture: 0.85). Sampling areas were six 240 µm×240 µm squares per retina, regularly spaced along the dorsal-ventral axis of retinal whole-mounts. Four retinal whole-mounts were used for each age of both WT and *rd1* mice (3 month-old and 18 month-old). Numbers of Thy1- and SMI32-positive cells were counted per grid square directly under microscope. Cell nuclei on the edge of the square were included if the nucleolus was visible. Numbers of DAPI–stained total cell nuclei were estimated in the same fashion. Endothelial cell nuclei were easily recognizable by their elongated shape and were not counted. The raw counts were then converted into cells/millimeter^2^.

To estimate the number of ipRGCs in both WT and *rd1* mice aged 3 and 18 months, we stained retinal whole-mounts with an antibody against melanopsin. The melanopsin antibody we employed here visualized effectively the M1 subtype of ipRGCs, while weakly staining of the M2 subtype of melanopsin cells. Some of M1 cells are conventionally placed with somata in the GCL, while the other M1 cells have somata displaced to the inner nuclear layer (INL). To determine the number of M1 ipRGCs, we counted the total numbers of conventionally placed and displaced M1 cells in six 500 µm×500 µm squares across the dorsal-ventral axis in optimally stained whole-mounted samples, using a 20× objective lens (numerical aperture: 0.75). Retinal margins and the area around the optic disk where staining appeared incomplete or was otherwise difficult to analyze were excluded.

To reconstruct the dendritic profiles of individual M1 ipRGCs, we traced M1 cells by live observation of immunostained cells in Neurolucida, using a 20× objective lens (numerical aperture: 0.75). By tracing all the dendrites from the optic fiber layer to the outermost immunopositive dendrites, we were able to reconstruct a virtually complete dendrite of these cells in the best-stained preparations with clearly resolvable immunopositive dendrites. For each age, roughly a half of reconstructed M1 cells were from the central retina, while the other half were from the peripheral retina. For the measurement of dendritic fields, a minimal convex polygon had been fitted around the dendritic profile of each fully reconstructed M1 cells. Similarly, we reconstructed SMI32-positive α ganglion cells in both the central and peripheral retinas in Neurolucida, using a 40× objective lens (numerical aperture: 0.85).

For the measurement of AAV-GFP transduced RGCs, we chose those well-isolated RGCs with an obvious axon. Two sets of images were collected for each cell. Corresponding z-stacks (1-µm steps) were taken for GFP (or neurobiotin injected) cells and DAPI using Metamorph (Universal Imaging, Downingtown, PA) to drive a focus motor (Ludl Electronic Products, Hawthorne, NY). A through-focus of the DAPI-stained nuclei was collected to measure the thickness of the inner plexiform layer (IPL) and determine the level of stratification of the GFP or neurobiotin injected cells. The level of stratification was defined as 0–100% from the border of the inner nuclear layer (INL) to the border of the ganglion cell layer (GCL). Off and On cells were stratified in the proximal (0–40%) and distal (40–100%) parts of the IPL, respectively. Cells were manually traced by using Neurolucida software (Microbrightfield, Colchester, VT). Metamorph stacks were opened in Neurolucida, and a reference point was made at the z-plane where the cell body was in focus. The cell body was outlined and then the dendrites and axon were traced by toggling up and down through the cell stack. At least three cells from each type were traced.

To evaluate whether alternations in the morphology occurred as the disease progresses, these morphological parameters were measured. (i) Dendritic field size: This parameter is measured as the area of the smallest convex polygon possible around the edge of the cell’s dendritic arbor when collapsed along the z-axis using the MetaMorph software. Dendritic field sizes were expressed as equivalent diameters, that is, the diameter of a circle of equal area. (ii) Total dendrite length: the total lengths of all the dendrites of individual RGC. (iii) Number of dendritic branches: all branches of total dendrites of individual RGC. (iv) Soma size: contour lines around each cell body used to calculate the area. Soma sizes were expressed as equivalent diameters. For each cell type, at each age, parameters were compared with those of corresponding RGCs from adult WT mouse retinas.

### Statistics

Data were represented as means ± SD. ANOVAs with Bonferroni’s and Dunnett’s *post hoc* tests for multiple comparisons were performed with Origin (OriginLab) and programs written in MATLAB (Mathworks) on full data sets to detect significant differences in the mean. A p value <0.05 was considered statistically significant.

## Results

The main goal in the present study was to assess alterations in morphological properties of RGCs in *rd1* mouse retinas following photoreceptor loss. In this end, we expressed GFP in RGCs to obtain an overview of the complete morphology of RGCs over a wide age range.

### Transduction of RGCs by an AAV-GFP Vector in *rd1* Eyes

**Figure 1:**
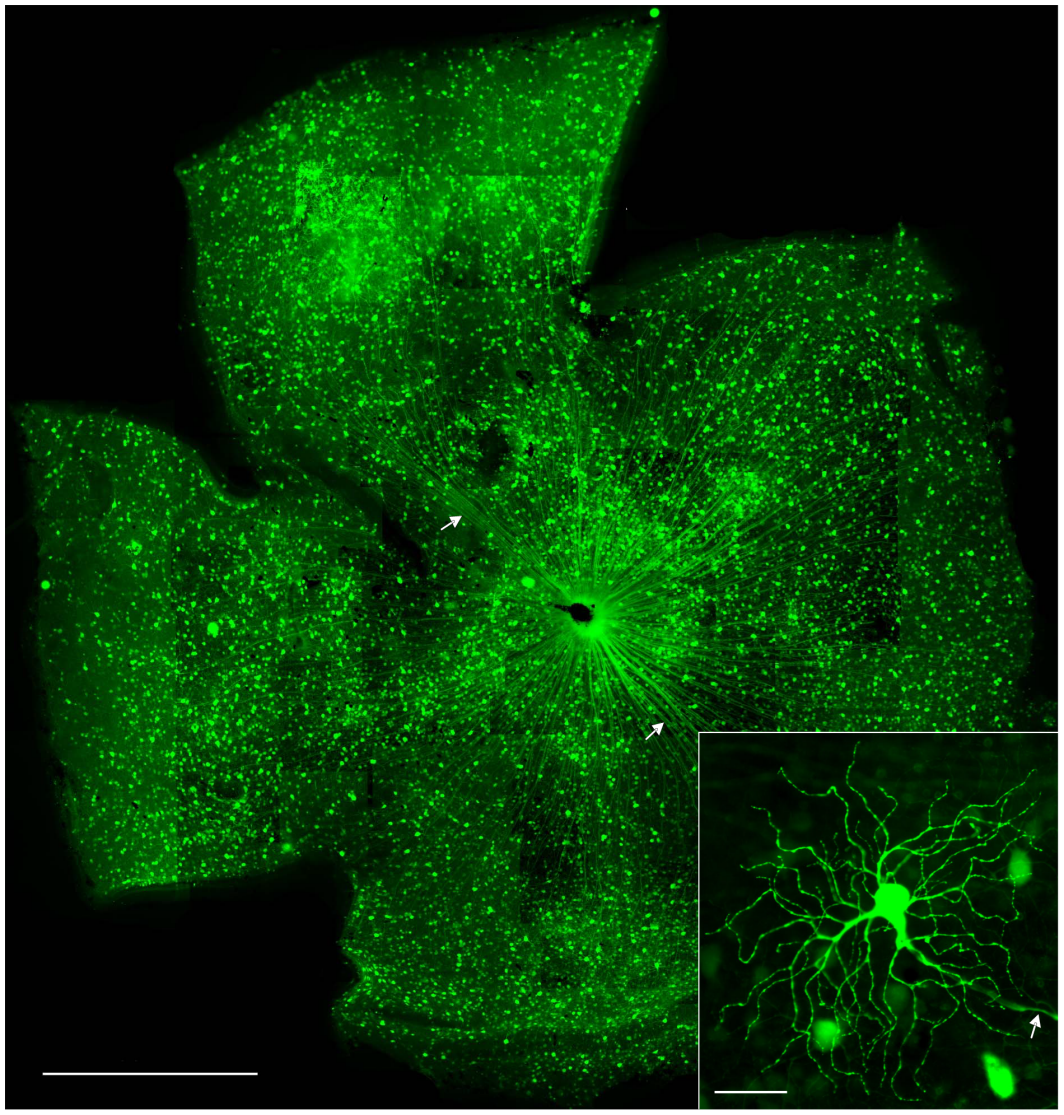
Transduction of retinal ganglion cells (RGCs) in *rd1* mice following intravitreal administration of an adeno-associated virus (AAV) vector carrying the gene for enhanced green fluorescent protein (EGFP). Representative retinal flat mount shows extensive GFP expression throughout the whole retina. Some GFP expressing cells have the size and dendritic morphology of RGCs and possess an axon leading from the cells to the optic disc (arrows), indicative of RGCs. The entire dendritic tree structure of some RGCs is clearly visualized by GFP expression (inset). Scale bars, 1 mm (100 µm in inset).

**Figure 2:**
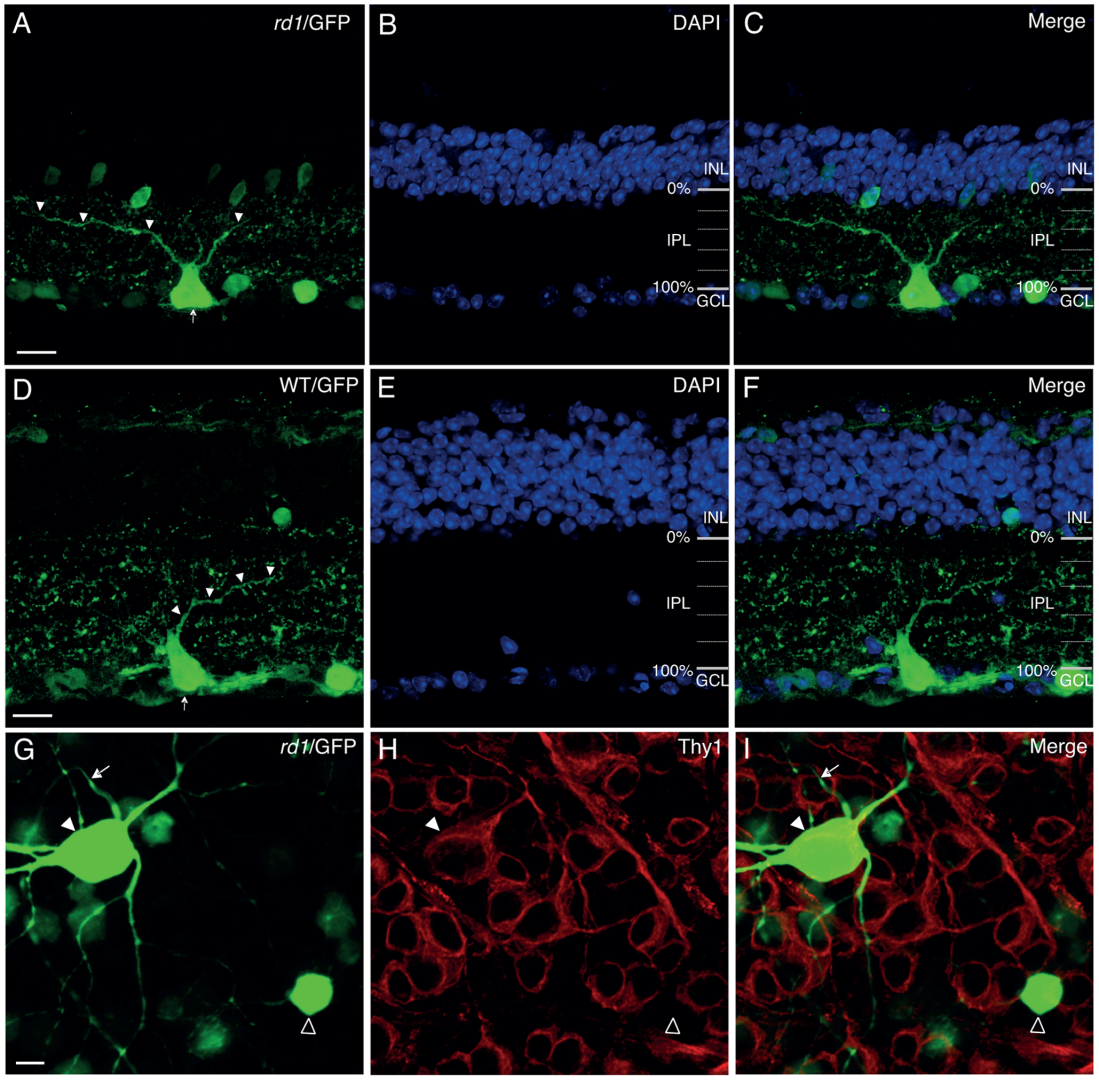
Determining the dendritic stratification level of RGCs in the IPL. **A**, One representative image of AAV-GFP transduced RGCs in a cross section of *rd1* retinas. In this illustration, the RGC has a big soma (arrow) and a broad dendritic tree (arrowheads). **B**, To define the stratification level of RGC dendrites in the IPL, we stained cell nuclei with diamidinophenylindole (DAPI). The INL and GCL delineate the proximal (0%) and distal (100%) boundaries of the IPL, respectively. **C**, Merged image shows that the dendrite of the RGC are centered at 25% of the IPL. **D**–**F**, One representative image of AAV-GFP transduced RGCs in a cross section of WT mouse retinas was shown as comparison. The transfection in WT was similar to what we observed in rd1 mice. **G**–**I**, To distinguish RGCs from diaplaced amacrine cells, AAV-GFP transduced retinas were co-labeled with an antiserum against Thy-1, a marker for all RGCs. Single image section was taken from the ganglion cell layer with focus on cell bodies, and the axon of one AAV-GFP transduced cell was out of focus (arrow). The transduced cell with an axon colocalized with Thy-1 (solid arrowhead), while another transduced cell without an axon did not colocalize with Thy-1 (open arrowhead). INL, inner nuclear layer; IPL, inner plexiform layer; GCL, ganglion cell layer. Scale bars, 20 µm in A–F, and 10 µm in G–I.

Adeno-associated virus (AAV) vectors have been used as a vehicle to deliver the gene of interest into eyes for the treatment of ocular diseases. In the present study, we explored the feasibility of using an AAV vector carrying a gene encoding enhanced green fluorescent protein (EGFP) as a reliable and relatively rapid method for revealing cell morphologies to greatly facilitate the identification and characterization of RGCs in *rd1* retinas. As expected, highly efficient transduction of RGCs was achieved in *rd1* retinas after four weeks of post-intravitreal injection of an AAV-EGFP vector (Fig. 1). RGCs are defined by the presence of an axon fiber stratifying in the nerve fiber layer and directed toward the optic disk. The visibility of axons of a large number of GFP-labeled cells confirmed that some of the transfected cells were RGCs (Fig. 1, arrows). The entire dendritic structures of some RGCs were clearly visualized by strong GFP expression (Fig. 1, inset). Thus, this method made it possible for us to survey different types of RGCs and followed the morphological changes of RGCs on a single-cell basis over time. RGCs are subdivided into two major functional classes: On ganglion cells and Off ganglion cells. The On ganglion cells that respond to light increments have axons terminating in the inner half of the inner plexiform layer (IPL), whereas the Off ganglion cells that respond to light decrements have axons which stratify in the outer half of the IPL [36]. One representative image of AAV-GFP transduced RGCs in a cross section of *rd1* retinas was shown in Figure 2. In this illustration, the RGC had a big soma (arrow) and a broad dendritic tree (arrowheads). To determine the dendritic stratification level of the GFP-labeled RGC in the IPL, DAPI-labeled nuclei were used to reveal nuclear layer boundaries between the inner nuclear layer (INL) and ganglion cell layer (GCL) (Fig. 2**B**). This cell was an Off RGC, whose dendrites were centered at 25% of the IPL, close to the INL (Fig. 2**C**). The AAV transduction in WT retinas was similar to what we observed in *rd1* retinas. One representative image of AAV-GFP transduced RGCs in a cross section of WT mouse retinas was shown as comparison (Figs. 2D–2F). To distinguish RGCs from amacrine cells, we stained AAV-GFP transduced retinas by using an antiserum against Thy-1, a marker for RGCs in the mouse [32], [35], [37]. Figure 2 shows one AAV-GFP transduced cell with an axon was co-labeled with Thy-1 (Fig. 2 G–I, solid arrowhead), while the other AAV-GFP transduced cell without an axon did not co-localize with Thy-1 (Fig. 2 G–I, open arrowhead), confirming that the cells with an axon were indeed RGCs and the cells without an axon were displaced amacrine cells.

### Visualization of Fine Morphology of RGCs by GFP Expression in *rd1* Retinas

**Figure 3:**
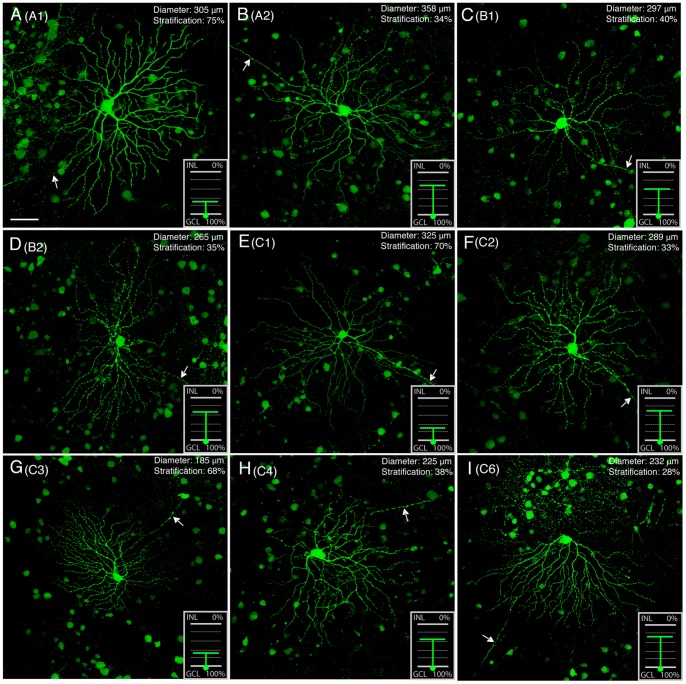
A large-scale survey of RGCs in ***rd1*** **retinas of 18 month-old.** Individual RGCs are visualized by GFP expression. Representations of nine types of RGCs are shown together with their dendritic diameters and stratification in the IPL. Numbers in parentheses correspond to the cell types distinguished by Sun et al. (2002). Insets show the dendritic stratification of RGCs in the IPL. In the inset, green circle shows the location of soma, and green line indicates dendrites. The dendrites of Off and On RGCs stratified in the proximal (0–40%) and distal (40–100%) parts of the IPL, respectively. Note that this representation shows the depth of the processes only and does not attempt to show their spread. Arrows indicate axons of individual representative RGCs. INL, inner nuclear layer; IPL, inner plexiform layer; GCL, ganglion cell layer. Scale bars, 50 µm.

The visualization of an individual RGC morphology by GFP expression allowed us to classify them and to evaluate in detail whether RGCs were morphologically intact over time in *rd1* retinas. By varying viral volume and incubation time, we obtained retinas with less transduced cells. We chose GFP-labeled cells with obvious axons for analysis [38]–[40]. More than a dozen different types of RGCs have been identified in the mouse retina [38]–[40], on the basis of their dendritic characteristics, such as dendritic shape, size and stratification level in the IPL and functional features. The most powerful ones among these criteria are the depth of arborization within the IPL and the branching pattern and breadth of the dendritic arbor. These three criteria were applied to classify RGCs in our samples. For every GFP-expressing RGC, two series of images were collected. The first was a series, at 1µm intervals, through the GFP-expressing RGC. The second was the DAPI-labeled nuclei focused through the IPL at the same location. In total, we studied 65 RGCs, which were located in both the center and peripheral retinas and whose GFP- processes did not overlap with those of other RGCs nearby. The GFP transduced RGCs we encountered were diverse in their morphology. Based on dendritic branching pattern, dendritic size and stratification level in the IPL, these cells were grouped into 9 different categories and are summarized in Table 2. Since RGCs of same types from both the central and peripheral retinas differed little in soma sizes, dendritic sizes and other dendritic parameter measurements, and have been pooled together in analysis (p>0.05, one-way ANOVA analysis). Figure 3 shows micrographs of representative types of RGCs we encountered in the retina of 18 month-old *rd1* mice, which were classified using nomenclature of He’s group [40]. While the dendritic field sizes of four types of RGCs (A1, B2, C1 and C2) in *rd1* retinas are comparable to their counterparts in WT retinas, those of the other five types of RGCs are either slightly larger (A2 and B1) or smaller (C3, C4 and C6) than their counterparts in WT retinas (Table 2) [40]. However, these differences were minor and dendritic field sizes remained within the normal range (p>0.05, one-way ANOVA analysis). Moreover, the branching patterns of dendritic arbors appears identical to their counterparts in WT mice reported previously by He’s group [40]. In addition to dendritic tree sizes, the total dendritic length, and the total number of nodes were also obtained from Neurolucida tracings (Table 3). These parameters we chose were highly sensitive to alternations in the dendritic morphology. We selected three cells from each cell type for an analysis of the dendritic morphology. There were no significant differences in morphometric parameters between mutant and WT (p>0.05, one-way ANOVA analysis). We concluded that RGCs in the *rd1* retina showed a remarkable degree of morphological stability and retained their characteristic branching and stratification patterns long after the disruption of normal retinal architecture and function in the outer nuclear layer (ONL).

**Table 2:** Summary of RGC classification encountered in this study.

Measurement	Cell type (Group)									
		G1(A1)	G2(A2)	G3(B1)	G4(B2)	G5(C1)	G6(C2)	G7(C3)	G8(C4)	G9(C6)
DF diameter Mean(µm) ± SD	RD1	309±64	312±45	251±31	168±33	260±71	274±52	271±84	190±43	209±75
	WT	317±47	301±53	235±45	152±46	288±59	262±43	305±65	222±57	232±54
Soma diameter Mean(µm) ± SD	RD1	21±5	23±3	18±5	15±4	19±4	18±3	16±3	17±2	15±3
	WT	20±6	23±5	19±7	14±7	20±6	18±4	15±6	17±5	16±4
Stratification (%)Mean ± SD	RD1	74±13	34±9	40±6	35±8	65±14	33±5	67±11	37±7	29±10
	WT	76±12	35±8	41±8	34±6	67±8	30±7	70±13	34±6	31±9
No. of cells	RD1	7	11	6	8	10	7	4	5	8
	WT	4	3	3	3	3	3	3	3	3

**Table 3:** Measurements of morphological parameters.

Measurement	Cell type (Group)						
		G1(A1)	G2(A2)	G3(B1)	G4(B2)	G5(C1)	G6(C2)
Total dendritic LengthMean (µm) ± SD	RD1	5501.2±280.5	5433.6±301.2	4984.4±310.6	4593.5±293.3	4389.4±256.5	3912.4±331.8
	WT	5763.4±351.8	5195.6±284.7	4678.8±413.8	4885.8±375.6	4003.6±327.4	4221.2±298.7
No of nodes Mean ± SD	RD1	40.3±4.6	39.6±3.5	69.8±8.7	55.2±8.4	39.5±4.2	34.9±2.6
	WT	38.7±3.7	36.3±2.6	71.3±9.6	48.3±6.8	35.8±5.6	37.3±5.4
No. of cells	RD1	3	3	3	3	3	3
	WT	3	3	3	3	3	3
		**G7(C3)**	**G8(C4)**	**G9(C6)**	**α**	**M1**	
Total dendritic Length.Mean (µm) ± SD	RD1	3583.5±145.5	3144.6±171.3	2884.4±210.5	5693.5±293.3	4003.5±285.6	
	WT	3773.4±151.6	2995.2±181.8	3002.8±163.8	5885.8±375.6	4130.8±325.3	
No of nodes Mean ± SD	RD1	56.4±5.5	42.4±3.5	32.3±3.4	34.8±3.9	25.6±4.4	
	WT	60.3±6.2	39.7±5.6	36.6±3.4	38.4±3.2	21.8±2.8	
No. of cells	RD1	3	3	3	3	3	
	WT	3	3	3	3	3	

### Visualization of Fine Morphology of RGCs by Neurobiotin Injections in *rd1* Retinas

**Figure 4:**
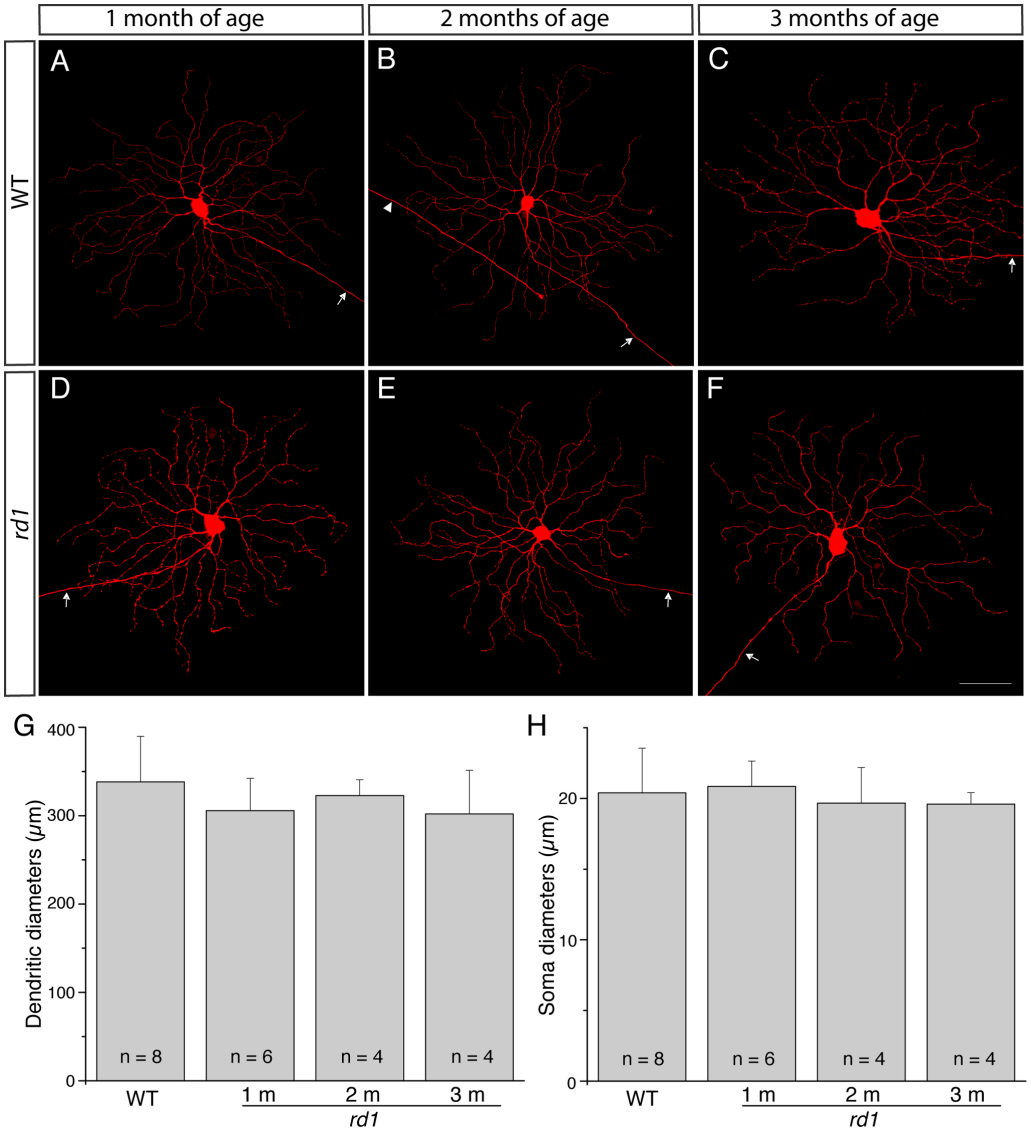
Representative A2 RGCs classified using nomenclature of Sun et al. (2002), from both WT and *rd1* mice of different ages. **A–F,** A2 RGCs were injected with neurobiotin, stained with fluorochrome-conjugated streptavidin, and reconstructed from mosaics of confocal Z-series. Arrows indicate axonal processes emerging from the somata of the A2 RGCs. Arrowhead in **B** indicates an axon of another injected RGC passing through this A2 RGC. **G**: Histogram of mean dendritic field diameters of A2 RGCs. There was no significant difference in dendritic-field sizes of A2 RGCs between WT and *rd1* retinas (p>0.05, one-way ANOVA analysis). **H**: Histogram of mean soma diameters of A2 RGCs. There was no significant difference in soma sizes between WT and *rd1* retinas (p>0.05, one-way ANOVA analysis). Scale bars, 50 µm.

We next examined whether AAV vectors caused any extra changes to the morphology of RGCs in *rd1* retinas. To eliminate this possibility, we used an independent technique to reveal the RGC morphology. We penetrated the perikarya of individual RGCs with sharp electrodes in a superfused retina preparation and iontophoretically injected the intracellular tracer neurobiotin. The purpose of the neurobiotin injection was to confirm whether viral transduction introduced any extra changes to the morphology of RGCs, we thus did not attempt to expand the neurobiotin injection to replace the viral transduction. We took one type of RGCs as an example and compared cell bodies and dendritic sizes of RGCs with AAV-GFP transduced RGCs of identical types. To increase our chance to target population of one type, we thus injected RGCs with a big body size. Even so, we had encountered several types of RGCs with a big body size. 22 out of 60 injected RGCs were classified as A2 RGCs using nomenclature of He’s group [40], whose dendrites were centered at 33% of the IPL (33% ±5 mean ± SD), close to the INL. Figure 4 shows micrographs of representative A2 RGCs from both WT and *rd1* mice of three different ages. The branching patterns of the dendritic arbors of A2 RGCs in *rd1* mice (Fig. 4. D, E and F) appear identical to their counterparts in WT mice (Fig. 4. A, B and C). Both dendritic sizes and soma sizes of A2 RGCs were measured (Fig. 4, G and H). The size of dendritic arbors remained stable over time in *rd1* retinas (305.7±36.5 µm mean ± SD for 1 month, n = 6; 322.7±17.9 µm for 2 months, n = 4; 302.1±49.3 µm for 3 months, n = 4; p>0.05, one-way ANOVA analysis). Also, our estimates of A2 RGC dendritic field sizes in *rd1* mice across different ages were in line with those of A2 RGCs in WT mice (328.1±51.6 µm mean ± SD, n = 8; p>0.05, one-way ANOVA analysis), and were in good agreement with estimates of A2 RGC dendritic field sizes from viral transduction (312±45 µm mean ± SD, n = 11; p>0.05, one-way ANOVA analysis). Soma diameters of A2 cells also remained identical between WT and *rd1* mice (In WT mice: 20.4±3.1 µm mean ± SD, n = 8; In *rd1* mice: 20.9±1.7 µm for 1 month, n = 6; 19.6±2.5 µm for 2 months, n = 4; 19.6±0.8 µm for 3 months, n = 4; p>0.05, one-way ANOVA analysis) (Fig. 4H). In summary, these results eliminated the possibility that viral transduction would cause changes to the morphology of RGCs. Moreover, we confirmed that A2 RGCs in *rd1* mice developed a normal morphology and maintained their morphologies over time.

### Full Survival of Two Identified Populations of RGCs in the *rd1* Mice

**Figure 5:**
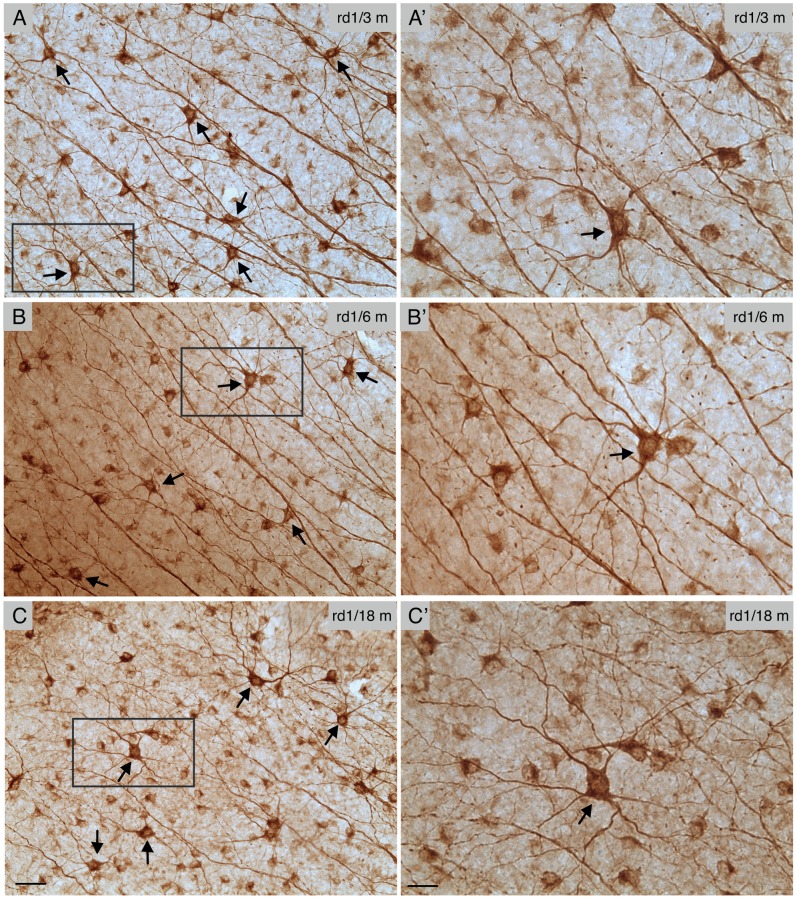
SMI-32 immunostaining in the *rd1* retina and the cell types it reveals. **A–C,** **** Antibody to SMI32 was reacted against flat mounted retinas of *rd1* mice of different ages, developed using DAB-immunohistochemistry. Arrows point to α ganglion cells. **A’–C’,** Highly magnified image from the boxed regions in **A–C**. Scale bars, 50 µm in A–C, and 25 µm in A’–C’.

**Figure 6:**
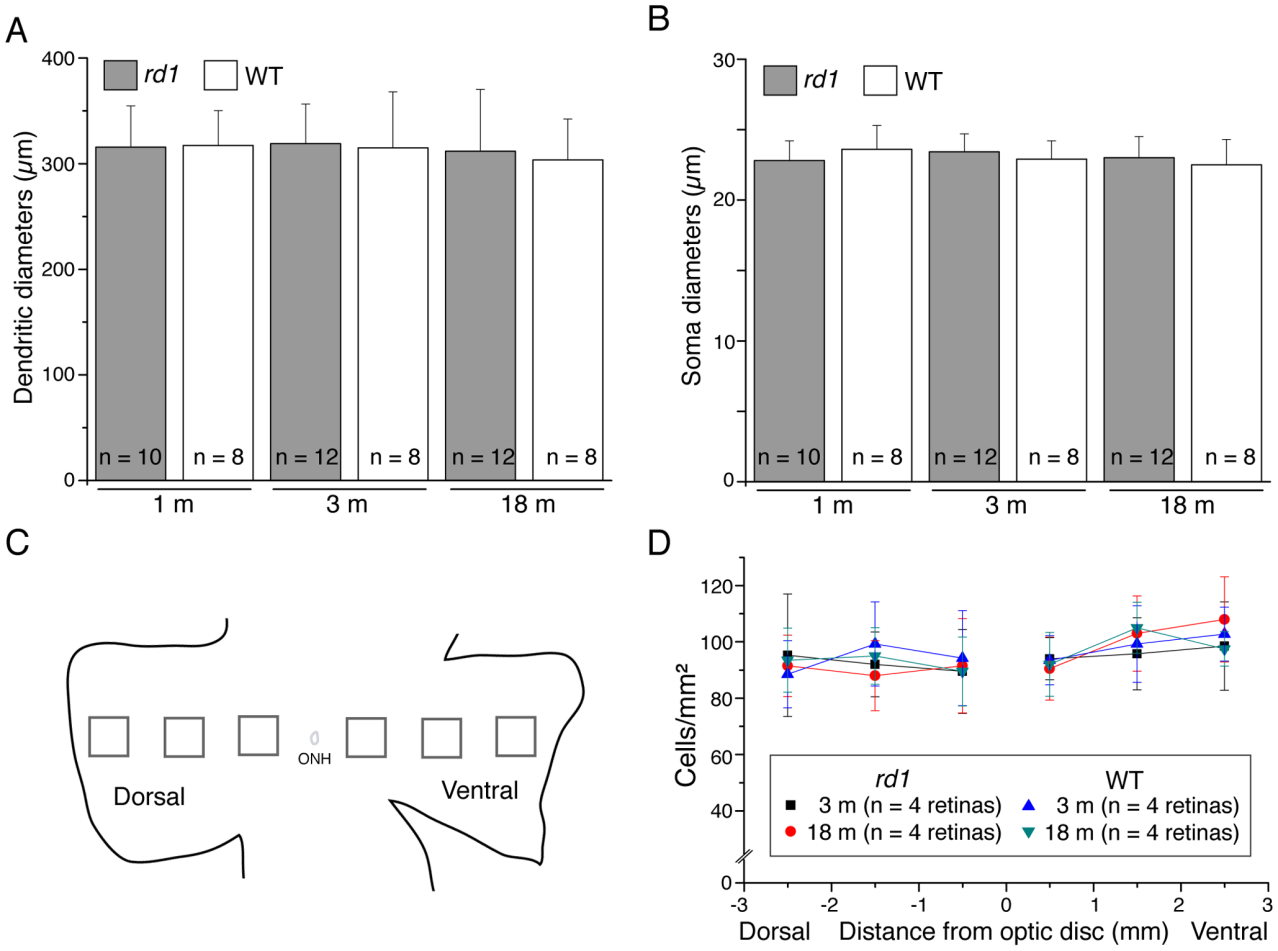
Somatic and dendritic-field sizes of SMI-32 positive α RGCs and their density distribution in the *rd1* retina. **A**: Histogram of mean dendritic field diameters of α ganglion cells stained by SMI-32. N indicates the number of α RGCs studied. **B**: Histogram of mean soma diameters of α ganglion cells stained by SMI-32. There was no significant difference in either dendritic-field sizes or soma sizes of α ganglion cells between WT and *rd1* retinas (p>0.05, one-way ANOVA analysis). **C**, The diagram illustrates six sampling areas, regularly spaced along the dorsal-ventral axis of retinal whole-mounts, were surveyed for all cell counting. ONH, optic nerve head. **D,** The graphs show the density distribution of SMI-32 positive RGCs from both WT and *rd1* mouse retinas at two different ages.

Photoreceptor degeneration shows a common central-to-peripheral progression pattern [26]–[29]. To investigate whether RGCs followed the same spatiotemporal pattern, we looked specifically at the populations of two types of RGCs using RGC-specific markers and assessed the cell morphology and number at different eccentricities. SMI32, which reacts with a non-phosphorylated epitope in neurofilament H, is relatively selective for α ganglion cells with large somata (∼ 25 µm), 4–5 thick primary dendrites, and large sparsely branched dendritic trees with a smooth character in the mouse retina [32], [34]. In addition, SMI32 stains several other types of RGCs with smaller dendritic diameters [32], [41]. The α ganglion cells, the largest ganglion cell of all, are reported to present in the mouse retina [38]–[40], [42]. SMI32-positive α ganglion cells were noted to have a similar appearance in their dendritic branching patterns across different ages of *rd1* mice (Fig. 5, arrows). There were also no significant differences in morphometric parameters between mutant and WT (p>0.05, one-way ANOVA analysis; Table 3). The dendritic sizes remained stable over a broad range of ages in *rd1* retinas [315.6±39.2 µm in diameter at 1 month of age (Mean ± SD, n = 10); 316.8±40.5 µm in diameter at 3 months of age (Mean ± SD, n = 12); 311.8±68.4 µm in diameter at 18 months of age (Mean ± SD, n = 12)] (Fig. 6A). In addition, our estimates of the dendritic field size of α ganglion cells in *rd1* mice were in line with those of α ganglion cells in the same aged WT mice (Fig. 6A, p>0.05, one-way ANOVA analysis). Similarly, SMI32-positive α ganglion cells differed little in soma size between WT and *rd1* mice (In WT mice: 23.6±1.7 µm mean ± SD for 1 month, n = 8; 22.9±1.3 µm for 3 months, n = 8; 22.5±1.8 µm for 18 months, n = 8; In *rd1* mice: 22.8±1.4 µm for 1 month, n = 10; 23.8±1.4 µm for 3 months, n = 12; 23.0±1.5 µm for 18 months, n = 12) (p>0.05, one-way ANOVA analysis) (Fig. 6B).

To assess a possible cell loss, we counted SMI-32-labeled α ganglion cells in *rd1* retinas at two different ages, which represented an early stage (3 months of age) and an advanced stage (18 months of age) of retinal degeneration (Fig. 6D). On average, no significant difference in the density of SMI32-labeled α ganglion cells was detected between these two ages across the retina (p>0.05, one-way ANOVA analysis). In addition, we did not observe a big drop in the cell density of SMI32-labeled α ganglion cells in the central retina with age (Fig. 6D), indicating that the central-to-peripheral progression of photoreceptor loss does not seem to have a detrimental effect on the population of α ganglion cells for up to 1.5 years of age. Moreover, our estimates of the average density of SMI32-labeled α ganglion cells in *rd1* retinas are in good agreement with those of SMI32-labeled cells in WT retinas of the same age (p>0.05, one-way ANOVA analysis) (Fig. 6D).

**Figure 7:**
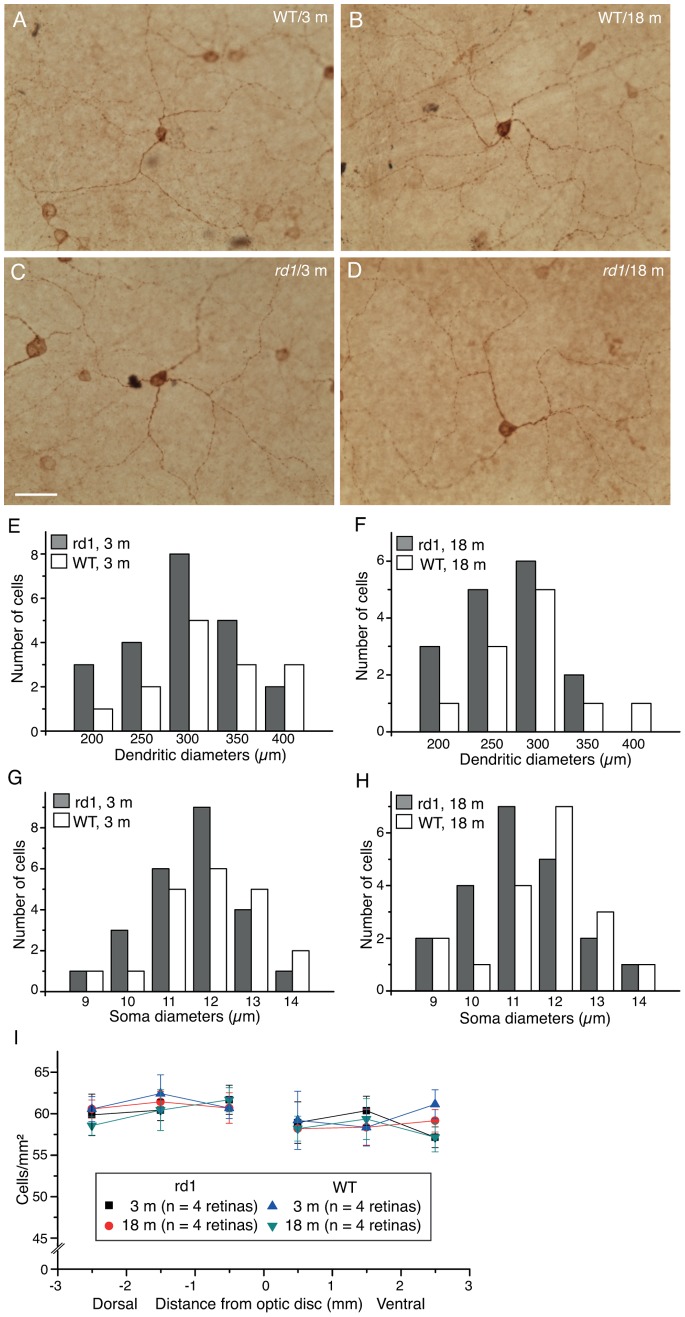
Intrinsically photosensitive RGCs (ipRGC) in the mouse retina. **A–D,** An antibody to melanopsin was reacted against flat mounted retinas and developed using DAB-immunohistochemistry. Scale bar, 50 µm. **E–F**: Histograms show the distribution of the dendritic field diameters of M1 cells in WT and *rd1* retinas at 3 month-old (**E**) and 18 momth-old (**F**). G–H: Histograms show the distribution of the soma diameters of M1 cells in WT and *rd1* retinas at 3 month-old (**G**) and 18 momth-old (**H**). I, The graph shows the average densities of M1 cells across the dorso-ventral axe from both WT and *rd1* mouse retinas at two different ages.

Melanopsin, the most recently discovered photopigment, is expressed in several subtypes of RGCs [33], [43]–[45]. These ganglion cells are intrinsically photosensitive (ipRGC) and mediate a broad range of physiological responses, such as photoentrainment of the circadian clock, light regulation of sleep, and the pupillary light reflex. An antiserum against melanopsin protein, which primarily labels M1 ipRGCs [33], [43], was applied to flat mounted retinas (Fig. 7 A–D). The M1 cells have small somata, and are composed of few, long processes of coarse caliber, which stratify exclusively in the outer margin of the IPL. The M1 ipRGCs from the retinas of either WT or *rd1* mice at different ages were morphologically indistinguishable on the basis of their dendritic branching patterns and the extent of the dendritic field (Fig. 7 A–D). The visual observation was confirmed by the comparisons of several morphometric parameters between *rd1* and WT (p>0.05, one-way ANOVA analysis) (Table 3). Moreover, the average dendritic diameters of M1 cells were very similar between *rd1* mice and WT mice of the same age (Fig. 7 E–F, p>0.05, one-way ANOVA analysis). Similarly, soma sizes of individual M1 cells differed little in their distributions between WT and *rd1* mice at both 3 and 18 months of age in our sample (Fig. 7 G–H; In WT mice: 13.1±1.5 µm mean ± SD for 3 months, n = 20; 13.0±1.1 µm for 18 months, n = 18. In *rd1* mice: 12.6±1.2 µm for 3 months, n = 24; 12.7±1.4 µm for 18 months, n = 21). No significant changes were observed in average soma diameters between *rd1* mice and WT mice of same ages (p>0.05, one-way ANOVA analysis).

To quantify the number of M1 cells, we examined four optimally stained whole-mounted samples from both *rd1* and WT mice. The somas of M1 cells were brightly labeled by the melanopsin antibody we used (ABR PA1-780) (Fig. 7, A-D) [32]. It thus was not difficult for us to classify and count M1 cells directly under microscope. Both conventionally placed and displaced M1 cells in six squares across the dorsal-ventral axis of retinal surface were determined (Fig. 7I). In WT retinas, the average densities of M1 cells were more or less uniform across the dorsal-ventral meridian, with a little higher density in the dorsal retina (61.2±1.7 cells/mm^2^ in the dorsal vs 59.6±2.5 cells/mm^2^ in the ventral at 3 month-old; 60.2±1.6 cells/mm^2^ in the dorsal vs 58.2±1.9 cells/mm^2^ in the ventral at 18 month-old; Fig. 7I). Similar numbers were observed in four 5-mon-old and four 18-mon-old *rd1* mouse retinas (Fig. 7I). The densities of M1 cells in *rd1* retinas were in good agreement with estimates from WT retinas of same age (p>0.05, one-way ANOVA analysis).

Taken together, our observations suggest that photoreceptor loss did not appear to cause any significant change in both the morphology and the cell number of two types of RGCs in *rd1* retinas, SMI32-positive α ganglion cells and M1 ipRGCs, for at least up to 1.5 years of age.

### Full Survival of Retinal Neurons in the RGC Layer of *rd1* Mice

**Figure 8:**
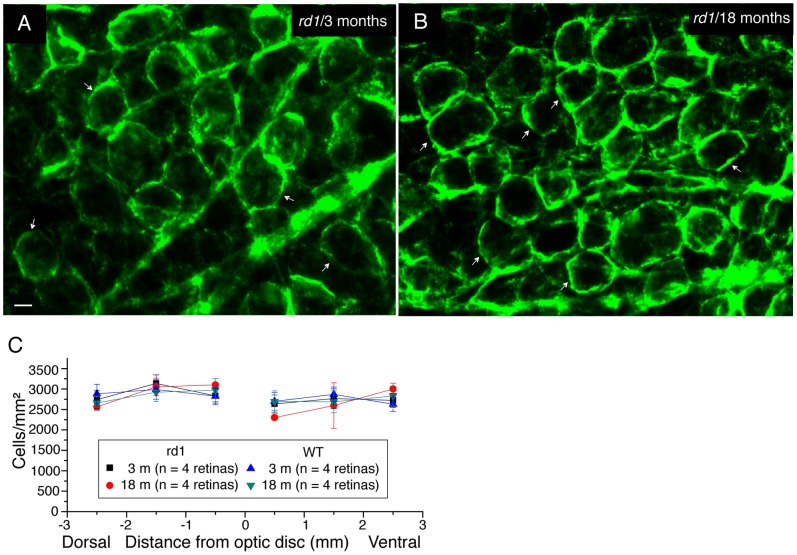
RGC Populations in *rd1* mouse retinas. **A–B,** RGCs were revealed by using an antiserum against Thy-1, a marker for all RGCs in the mouse retina. Two representative images were taken from *rd1* retinas at the age of 3 months (**A**) and 18 months (**B**). Thy-1 is a surface glycoprotein uniquely expressed in RGC’s membrane (Arrows). Scale bar, 10 µm. **C,** Quantification of Thy-1 positive cells in the ganglion cell layer of both WT and *rd1* retinas at two different ages. Values are mean ± SD, with n indicating the number of retinas counted.

To evaluate possible cell number deduction in other types of RGCs, we quantified the number of RGCs that were immunoreactive for Thy-1, a marker for all RGC populations in the mouse retina [32], [35], [37]. The density of Thy-1 positive cells at 18 month-old *rd1* mice appeared identical to that of Thy-1 positive RGCs in the retina of 3 month-old *rd1* mice, by visual inspection (Fig. 8, A and B). The quantification of Thy-1 positive RGCs confirmed our impression (Fig. 8C). The average densities of Thy-1 positive RGCs in *rd1* mice did not change over time, and was almost identical to each other between the early stage (3 month-old) and late stage of retinal degeneration (18 month-old) (p>0.05, one-way ANOVA analysis). Moreover, the average density of Thy-1 positive RGCs in *rd1* mice was close to that of Thy-1 positive RGCs in WT mice of same age (In WT: 2814.7±208.7 cells/mm^2^ at 3 month-old, 2794.2±240.9 cells/mm^2^ at 18 month-old; In *rd1*∶2804.6±206.6 cells/mm^2^ at 3 month-old, 2768.7±208.7 cells/mm^2^ at 18 month-old. p>0.05, one-way ANOVA analysis) (Fig. 8C). In summary, our data suggested that the RGC populations probably remained intact in the *rd1* retina for at least up to 18 months of age in our sample.

**Figure 9:**
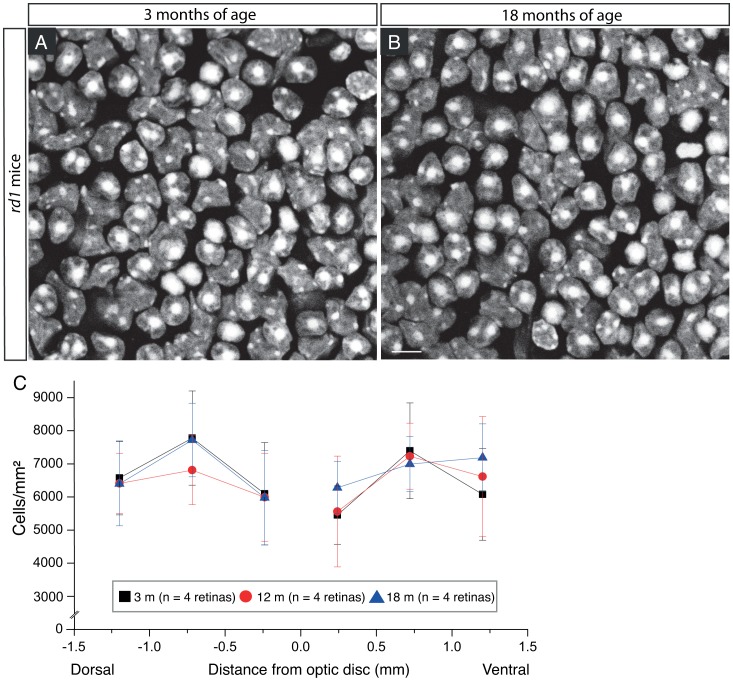
Cell populations in the ganglion cell layer of *rd1* mouse retinas. **A–B,** Images of DAPI labeled cells in the ganglion cell layer were taken from the retinal regions located 1 mm superior to the center of the optic nerve head at two different ages. Scale bar, 10 µm. **C,** Quantification of DAPI labeled cells in the ganglion cell layer of *rd1* mice at three different ages. Values are mean ± SD, with n indicating the number of retinas counted.

In addition to RGCs, the retinal ganglion cell layer (GCL) also contains a substantial number of displaced amacrine cells [46], [47]. To determine whether the number of displaced amacrine cells was affected by photoreceptor degeneration, we stained retinal nuclei in the GCL with diamidinophenylindole (DAPI). Confocal images of DAPI labeled cells were taken from the region 1mm superior to the center of the optic nerve with focus at the GCL (Fig. 9, A and B). The two images from two different ages of *rd1* mice (3 month-old and 18 month-old) appeared very similar in term of cell density. As expected, the quantification of DAPI labeled cells showed no significant differences in the cell average density among the three age groups (Fig. 9C; p>0.05, one-way ANOVA analysis). Moreover, the average densities of DAPI labeled cells in *rd1* retinas were also in good agreement with estimates of the average densities from WT retinas of the same age (In WT: 6651.7±1108.5 cells/mm^2^ at 3 month-old, n = 3 retinas; 6394.2±1240.6 cells/mm^2^ at 12 month-old, n = 3 retinas; 6694.2±1408.9 cells/mm^2^ at 18 month-old, n = 3 retinas. p>0.05, one-way ANOVA analysis). Our data thus indicated that both ganglion cells and displaced amacrine cell populations in the GCL were not obviously affected by photoreceptor degeneration for up to 1.5 years of age.

## Discussion

In present study, we explored the feasibility of using an AAV vector carrying a gene encoding enhanced green fluorescent protein (EGFP) as a reliable and relatively rapid method for revealing fine RGC morphologies. With this method, we report here an initial survey of RGCs in *rd1* mouse retinas. We found that ganglion cell populations and their fine dendritic geometry remained intact well beyond the complete loss of photoreceptors for at least up to 1.5 year of age. It should be noted, however, that the accuracy of our observations in the present study might be limited by the small size of the sample and possible sampling bias, so it remains unclear whether subtle remodelling might have occurred among RGCs that is not detected by our experimental strategy.

### Maintenance of Dendritic Structures and Populations of RGCs in *rd1* Retinas

The temporal overlap between the early onset and rapidly progressing degeneration of photoreceptors and retinal synaptogenesis may disrupt dendritic development and stratification in neurons downstream of photoreceptors, resulting in abnormal dendritic phenotypes in the *rd1* mouse model of retinal degeneration. Indeed, neurons in the inner retina either fail to develop normal morphology, such as rod bipolar cells and horizontal cells [13], or undergo dendritic regression after morphological maturity such as cone bipolar cells, during and after photoreceptor degeneration [30]. To evaluate the impact of photoreceptor loss on the populations of RGCs in *rd1* mice, we revealed the dendritic processes of individual RGCs using an AAV-GFP vector, and tracked the possible changes in cellular architecture longitudinally. In contrast to the behavior of bipolar and horizontal cells, RGCs, which are two synapses away from photoreceptors, were especially resistant to the photoreceptor degeneration. The characteristic branching, geometry and stratification patterns of individual RGCs of nine different types evaluated in present study were largely maintained long after the complete loss of photoreceptors. These observations are consistent with a recent finding of the morphological preservation of a few of On and Off RGCs with the largest soma diameters in the *rd1* retina [24], as well as with the observation in another mouse model of RP, *rd10* mouse, which carries a missense mutation in the *Pde6b* gene and has a slower course of degeneration [23]. Our findings are also supported by the physiological finding that RGCs in *rd1* mice maintain a high degree of functional stability even into late stage retinal degeneration [24]. In addition, qualification revealed no cell number deduction in the populations of either two identified types of RGCs or every type of RGCs in the *rd*1 mice for as old as 18 months. During degeneration, bipolar cells and horizontal cells, the second-order retinal neurons, undergo massive regression of dendrites and subsequent cell death [13], [48], whereas RGCs appear to retain their morphology and cell populations for up to the age of 18 month in the same retinas, suggesting a possible minor role photoreceptors play in the development and maintenance of RGCs.

For the estimates of the number of survival RGCs in the animal models of RP, some studies have reported cell loss in *rd1* mice and P23H and RCS rats [17]–[20], while others have not observed any cell loss in *rd1* and rd10 mice [21]–[23]. These differences may reflect the difficulty of calculating absolute number of RGCs before an antibody, which could label every RGCs, becomes available. After having applied several best available antibodies to label RGCs, we also detected no change in the cell number of major RGC types. On the other hand, we observed the maintenance of the dendritic diameters in several major types of RGCs for up to 18 months of old in contrast to one previous study in the same line [21]. It should be noted, however, that only three types of RGCs were investigated in both studies. Type C2, M1 ipRGC and α RGC were observed to maintain normal dendritic arbors in our study, but the same three types of RGCs were found to have undersized dendritic arbors in the Damiani et al (2012) study. The cause of the discrepancy is unclear, but it could due to different animal genetic backgrounds and experimental strategies. We monitored morphological changes in RGCs directly in *rd1* mice, while Damiani et al (2012) studied RGCs in Thy1-GFP/*rd1* mice, in which Thy1-GFP mice were backcrossed onto the *rd1* mouse background for several generations. Another possibility is different classification criteria used in two studies. For instance, the dendritic stratification level in the IPL is one of the most important criteria used in our study (Fig. 2C). However, in some cases, it appeared that RGCs whose dendrites stratified in different depths within the IPL were grouped as one type (their Figs. 8 and 9). Moreover, M1 ipRGCs have a wide range of dendritic diameters ranging from 200 µm to 400 µm in mouse retinas [33], [49]. While it appears that the distribution of M1 cells was slightly shifted toward smaller dendritic field sizes in the *rd1* compared to the WT, the mean equivalent dendritic field diameters were statistically indistinguishable between WT and *rd1* mice. The same is true for the soma sizes. Furthermore, SMI32 antibody primarily labeled α RGCs, but also stained several other types of RGCs with smaller dendritic diameters in mouse retinas (Fig. 5, A–C’) [32], [41]. Only SMI32 positive α RGCs were analyzed in the present study, (Fig. 5, A’–C’). The other types of SMI positive RGCs were totally excluded. Taken together, our data suggest that the degeneration occurs in the outer retina appears to have little effect on the survival of RGCs in the inner retina, suggesting the central visual targets of RGCs seem to have strong influences on the survival of RGCs. However, it remains unclear whether RGCs will continue to maintain their normal morphologies well beyond 18 months of age in *rd1* retinas.

### Mechanisms for RGC Survival

Mechanisms leading to RGC survival are less clear. However, it seems that RGC survival is dependent on a combination of intrinsic activity and extrinsic target-derived trophic factors. Spontaneous activity of RGCs is one potential mechanism that leads to survival of RGCs. The rhythmic activity in cells has been proposed to represent an intrinsic mechanism for keeping cells alive in the retina without normal synaptic transmission [24], [48]. RGCs do not become quiescent after the loss of photoreceptors in *rd1* retinas. Instead, RGCs have been reported to sustain a spontaneous hyperactivity even the animal is going blind for an extended period in *rd1* retina [24], [50], [51]. Moreover, the ipRGCs, which express the photosensitive melanopsin protein, are only cells that respond to visual stimulation in the blind retinas. IpRGCs have recently been found to strongly resist to neurodegeneration and show a 3-fold increase in survival rate compared to the conventional non-ipRGCs after optic nerve transection [52] or in a chronic ocular hypertension model [53], [54]. However, ipRGCs and non-ipRGCs were found to be equally susceptible to ocular hypertension in a rat glaucoma model of chronic ocular hypertension [55] and DBA/2J [56], a mouse model of inherited glaucoma, in which the optic nerves were damaged. Taken together, intrinsic activities alone apparently do not seem to be sufficient to keep RGC survival for long time in these chronic disease conditions.

The central targets of RGCs, on the other hand, seem to exert strongly influence on the health and survival of a target cell population through the delivery of neurotrophic factors. Loss of neurotrophic factors has been hypothesized to contribute to the pathogenesis of several neurodegenerative disorders [57]–[59]. In neonatal rats, ablation of the superior colliculus, which is one of the central targets for RGCs, causes massive retrograde RGC death [60]. In addition, in adult rats, more than 80% of the retinal ganglion cells are lost within 2 weeks of optic nerve (ON) transection near the eye [61], [62], even though photoreceptors and bipolar cells in the same retina are normal and healthy. It is presumed that the trophic support that is normally provided by their distant targets maintains the function and consequently the viability of RGCs in the retina. Indeed, brain-derived neurotrophic factor (BDNF) produced by the superior colliculus (or the lateral geniculate nucleus in higher mammals) is an important survival factor for RGCs during early development and in adult life [63], [64]. On the other hand, reduced delivery of BDNF leads to vulnerability in GABAergic medium spiny neurons in the caudate and putamen in Huntington’s diseases, and eventually induces age-related degeneration of these neurons [65]. RGCs in the *rd1* retina retain their synaptic connections with higher visual centers [66]–[69]. Therefore, continuous delivery of pro-survival neurotrophic factors from visual centers is probably a key factor contributing to the survival of RGCs in *rd1* mice.

Collectively, our data suggested that ganglion cells, the only output cells of the retina, are especially resistant to photoreceptor degeneration, despite dramatic degeneration-induced disruption of normal retinal architecture and function in the outer retina, indicating that the ganglion cell population might be an attractive target in treating vision loss.
